# Mifepristone and misoprostol versus misoprostol alone for uterine evacuation after early pregnancy failure: study protocol for a randomized double blinded placebo-controlled comparison (Triple M Trial)

**DOI:** 10.1186/s12884-019-2497-y

**Published:** 2019-11-27

**Authors:** Joyce van den Berg, Charlotte C. Hamel, Marcus P. Snijders, Sjors F. Coppus, Frank P. Vandenbussche

**Affiliations:** 1 Department of Obstetrics and Gynaecology, Canisius-Wilhelmina Hospital, Postbus 9015, Nijmegen, GS 6500 The Netherlands; 2 Department of Obstetrics and Gynaecology, Radboud University Medical Centre, Geert Grooteplein Zuid 10, Nijmegen, GA 6525 The Netherlands; 3 Department of Obstetrics and Gynaecology, Maxima Medical Centre, Veldhoven, De Run 4600, Veldhoven, DB 5504 The Netherlands

**Keywords:** Early pregnancy failure, Miscarriage, Missed abortion, Mifepristone, Misoprostol

## Abstract

**Background:**

Early pregnancy failure (EPF) is a common complication of pregnancy. If women do not abort spontaneously, they will undergo medical or surgical treatment in order to remove the products of conception from the uterus. Curettage, although highly effective, is associated with a risk of complications; medical treatment with misoprostol is a safe and less expensive alternative. Unfortunately, after 1 week of expectant management in case of EPF, medical treatment with misoprostol has a complete evacuation rate of approximately 50%. Misoprostol treatment results may be improved by pre-treatment with mifepristone; its effectiveness has already been proven for other indications of pregnancy termination.

This study will test the hypothesis that, in EPF, the sequential combination of mifepristone with misoprostol is superior to the use of misoprostol alone in terms of complete evacuation (primary outcome), patient satisfaction, complications, side effects and costs (secondary outcomes).

**Methods:**

The trial will be performed multi-centred, prospectively, two-armed, randomised, double-blinded and placebo-controlled. Women with confirmed EPF by ultrasonography (6–14 weeks), managed expectantly for at least 1 week, can be included and randomised to pre-treatment with oral mifepristone (600 mg) or oral placebo (identical in appearance). Randomisation will take place after receiving written consent to participate. In both arms pre-treatment will be followed by oral misoprostol, which will start 36–48 h later consisting of two doses 400 μg (4 hrs apart), repeated after 24 h if no tissue is lost. Four hundred sixty-four women will be randomised in a 1:1 ratio, stratified by centre.

Ultrasonography 2 weeks after treatment will determine short term treatment effect. When the gestational sac is expulsed, expectant management is advised until 6 weeks after treatment when the definitive primary endpoint, complete or incomplete evacuation, will be determined. A sonographic endometrial thickness < 15 mm using only the allocated therapy by randomisation is considered as successful treatment. Secondary outcome measures (patient satisfaction, complications, side effects and costs) will be registered using a case report form, patient diary and validated questionnaires (Short Form 36, EuroQol-VAS, Client Satisfaction Questionnaire, iMTA Productivity Cost Questionnaire).

**Discussion:**

This trial will answer the question if, in case of EPF, after at least 1 week of expectant management, sequential treatment with mifepristone and misoprostol is more effective than misoprostol alone to achieve complete evacuation of the products of conception.

**Trial registration:**

Clinicaltrials.gov (d.d. 02-07-2017): NCT03212352.

Trialregister.nl (d.d. 03-07-2017): NTR6550.

EudraCT number (d.d. 07-08-2017): 2017–002694-19.

File number Commisie Mensgebonden Onderzoek (d.d. 07-08-2017): NL 62449.091.17.

## Background

In the Netherlands, every year more than 10.000 women with early pregnancy failure (EPF) undergo surgical or medical treatment in order to remove the products of conception from the uterus [1]. For many years, surgical treatment (dilatation and curettage, D&C) has been standard treatment [2]. However, D&C is associated with risks of complications (uterine perforation, pelvic infection, excessive bleeding, anaesthesia, intra-uterine adhesions, cervical injury or cervical insufficiency in following pregnancies) and high costs [3–7].

The Royal College of Obstetricians and Gynaecologists as well as the “American College of Obstetricians and Gynecologists” recommend medical methods as a safe, effective and acceptable alternative (evidence level A) [8, 9]. Misoprostol is used off-label for several obstetric and gynaecologic indications, including EPF, due to uterotonic properties leading to ripening and dilatation of the cervix and myometrial contractions [10]. For medical treatment, the International Federation of Gynecology and Obstetrics (FIGO) recommends the prescription of two doses misoprostol 800 μg administered vaginally (3 hrs apart) or two doses misoprostol 600 μg sublingual (3 hrs apart) [11, 12].

A minimum of 1 week of expectant management, which is common practice in the Netherlands results in spontaneous complete abortion rates of 50% [2, 13]. Unfortunately, if no spontaneous abortion has occurred after this week, and misoprostol treatment is applied, this remains unsuccessful in approximately half of the women. They still have to undergo surgical treatment and thus may be exposed to the risks of complications associated with D&C [2, 14–16].

Mifepristone is a progesterone antagonist and its administration during pregnancy increases the production of endogenous prostaglandin by the endometrium, the sensitivity of the gravid uterus to exogenous prostaglandin, the contractility of the myometrium, and cervical softening and dilatation [17, 18]. For other indications, such as labour induction in case of fetal death after the first trimester, and also for medical termination of vital pregnancy (medical abortion), the sequential combination of mifepristone followed by misoprostol has been shown superior to the use of misoprostol alone [19, 20]. So, it appears reasonable to consider mifepristone with misoprostol to be superior to misoprostol alone in case of EPF (non-vital pregnancy in the first trimester).

Several groups have been investigating the sequential combination of mifepristone with misoprostol in EPF, and reported success rates of 66–93% without serious adverse events [7, 11, 14, 21–28]. Unfortunately, these studies were small and flawed by different inclusion criteria and treatment regimens or retrospective study design [14]. A double-blinded pilot *pilot* study performed by our research group including women with EPF between 6 and 14 weeks of gestation after a minimum of 1 week of expectant management, showed a success rate of 68,4% (mifepristone + misoprostol, M&M) versus 40% (placebo + misoprostol). The need for second treatment, i.e. surgical intervention, was significantly lower in the M&M group as compared to the placebo group: 10,5% versus 50% respectively (*p* < 0.05) [29]. However, to develop evidence based treatment regimen, a sufficiently powered, randomised, double blinded, and placebo-controlled trial is urgently needed.

## Methods / design

### Study aim and design

The aim of this study is to compare addition of mifepristone to the standard treatment with misoprostol in terms of complete evacuation of products of conception from the uterus, patient satisfaction, complications, side effects and costs. The trial will be performed multi-centred in the Netherlands and will be conducted prospectively, two-armed, randomised (1:1 ratio), placebo-controlled and double blinded. Participating hospitals can be district, teaching or third referral (academic) hospitals. Participants are followed in outpatient clinics; hospital admission follows only if medically necessary. Ethical approval to conduct the study was obtained at the regional medical-ethical commission (Commisie Mensgebonden Onderzoek Arnhem-Nijmegen).

### Participants and eligibility criteria

Women with a diagnosis of EPF between 6 and 14 weeks of gestation. EPF is diagnosed by transvaginal ultrasonography describing:

- A crown-rump length ≥ 6 mm and no cardiac activity OR
- A crown-rump length < 6 mm and no fetal growth at least 1 week later OR
- A gestational sac with absent embryonic pole for at least 1 week.

A minimum of 1 week of expectant management results in spontaneous complete abortion rates of 50%, and is common practice in the Netherlands [2, 13]. Therefore, women will only be included if a minimum of 1 week of expectant management has been implemented. Women will however be suitable for inclusion immediately if there is an obvious discrepancy of at least 1 week between the crown-rump length and the calendar gestational age. .

Exclusion criteria are age < 16 years, hemodynamic instability, sign of infection, incomplete miscarriage, contra-indications for mifepristone or misoprostol, potential interaction between study-medication and other medication, language barrier or the inability to give informed consent, a known clotting disorder or use of anticoagulants or known risk factors for, or presence of a, cardiovascular disease.

### Procedures, recruitment, randomisation, and collection of baseline data

Women visiting a hospital in case of EPF are identified and approached to participate in the trial by their treating physician. Trained staff will counsel patients, inform about the aims, methods, reasonable anticipated benefits and potential hazards of the study and hand out the patient information letter. Patients will also be informed about the off-label use of mifepristone and misoprostol. Participation is voluntary and patients may withdraw consent to participate at any time during the study. The investigator can decide to withdraw a subject from the study for urgent medical reasons. Baseline demographics, obstetric and medical history are recorded for all women at the time of randomisation using a case report form. After obtaining written informed consent, randomisation can be performed.

The Clinical Trial Unit of the Radboudumc will coordinate randomisation. Subjects will be randomised in a 1:1 ratio to mifepristone 600 mg oral or placebo using computerised randomisation tables. The randomisation will be conducted using block randomisation and stratified by hospital. After randomisation a unique study number will be assigned corresponding with a study package available in the participating centre containing the blinded study medication. The placebo and mifepristone tablets are identical in appearance so neither the patient nor the physician will know which product is taken. Only the pharmacy will know which medication or placebo the patient has received. Blinding, distribution and labelling of the study medication packages will be coordinated by the clinical trial unit in the Radboudumc (Nijmegen). A sealed list with the label codes will be available there in case of emergencies. These data will be disclosed to the principal investigators only after data on all outcome parameters have been collected for all patients. Regarding misoprostol, the treating physician will prescribe these tablets as usual, disposed by the patients own pharmacy.

### Interventions and follow-up

**Fig. 1:**
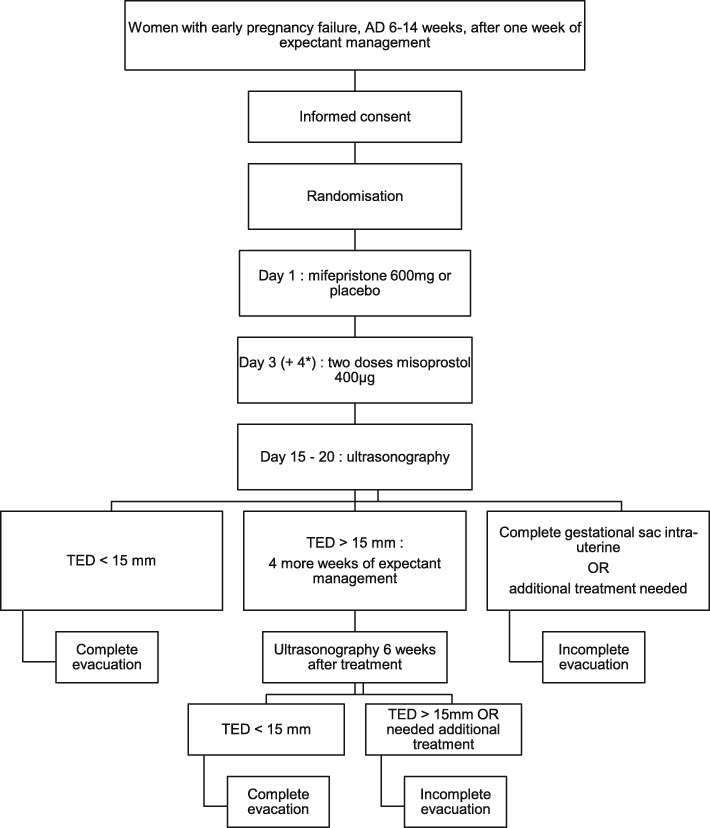
Flowchart of study procedures. *If no tissue is lost by day four, two more doses of misoprostol will be taken at day four

After informed consent and randomisation, each patient receives three (blinded) tablets containing 200 mg mifepristone each or placebo (day 1, Fig. 1). Apart from the study medication, management of participants will be similar in both groups. At day three (36–48 h later), two doses of misoprostol 400 μg orally (4 hrs apart) will be taken at home. If no tissue is lost by day four, two more doses of oral misoprostol 400 μg orally (4 hrs apart) will be taken at home. The administration of a second course of misoprostol, starting approximately 24 h after the first course, is common practice in the Netherlands. This doesn’t require an extra medical examination or ultrasound, but is based on the assessment regarding loss of tissue by the patient. The administration of misoprostol on day four will thus not be seen as a failure.

Regarding mifepristone, the World Health Organization advises mifepristone (200 mg) in combination with misoprostol in case of termination of a vital pregnancy in the first trimester [30]. Reasons for this lower dosage of mifepristone are not mentioned in this guideline; one could imagine it’s because of the, until recent, high costs of mifepristone in the context of low-resource countries. However, two phase 2 trials have shown that 600 mg mifepristone is superior to the 200 mg dose in terms of complete abortion in case of termination of a vital pregnancy (89% versus 63%) [31, 32]. A Cochrane Review included only one trial comparing low and high doses of mifepristone in case of medical abortion, and reported no significant difference in side-effects [20]. Furthermore, when considering other aspects of the expulsion of a pregnancy there is, maybe surprising, evidence that women receiving 600 mg mifepristone report a lower mean pain severity and even a lower prevalence of side-effects than the women receiving 200 mg mifepristone [33].

Concerning misoprostol, many different treatment regimens have been described with various routes of administration and doses. Up until 2014, 23 different treatment regimens (dosages and routes of administration) were used in the Netherlands, and in many hospitals even more than one treatment regimen existed simultaneously [34]. Several reviews conclude that research is still necessary to determine the most optimal treatment regimen [35, 36]. Vaginal application of misoprostol is widely accepted. However, oral misoprostol may be advised if combined with mifepristone, based on a significant lower infection rate in case of medical abortion after changing the regime of vaginal to oral administration [37]. When pharmacologically comparing oral and vaginal administration of misoprostol, oral misoprostol leads to a more rapid absorption and higher peak levels [38]. However, the mean time to expulsion was longer after oral intake of misoprostol compared to vaginal application [39, 40]. Clinical studies comparing oral and vaginal misoprostol have found increased satisfaction with the oral route because it is easy to use and avoids any unnecessary vaginal examinations [41, 42]. In our study protocol, the oral route is chosen because it appears equally effective compared to vaginal application, is easy to use, and makes for an increased patients satisfaction [41]. Gastrointestinal side effects are dose and interval dependent, higher doses and short intervals may lead to an increase in symptoms [38, 43]. Although one would suspect that oral misoprostol leads to more side effects due to higher peak concentrations, an equal incidence of vomiting, nausea, diarrhoea and fever was reported in a recent Cochrane review [44]. However, it should be mentioned that the quality of the included studies is low. In contrast to this recent Cochrane review concerning EPF, reviews including incomplete miscarriages or termination of vital pregnancies in the first trimester do report significantly more nausea and diarrhoea after oral misoprostol [20, 39].

Regarding effectiveness of misoprostol treatment, a Cochrane review reported that misoprostol 800 μg orally is equally effective compared to misoprostol 800 μg vaginally [11, 44]. A split dosage of misoprostol (two or three doses of 400 μg) has been reported to be similar in success rates as a protocol using 800 μg at once [20, 44]. Since a split-dose regimen is equally effective, but may lead to a lower incidence of side effects, we have chosen a split dose of misoprostol 400 μg. Thereby, if the first dose of misoprostol 400 μg leads to complete expulsion of the gestational sac, the second dose doesn’t have to be taken.

With regards to the follow-up of women receiving medical treatment, there are no clear internationally evidence based recommendations about the time frame and optimal diagnostic tool to define success. Ultrasonography seems to be of limited value in predicting the presence of intrauterine remnants. Besides, recent studies do not provide any clear evidence which endometrial thickness corresponds best to the presence of intrauterine pregnancy remnants [16, 45]. A study by Rulin et al. concludes that in case of a maximum anterior-posterior diameter of 15 mm or less, retained products are less likely to be confirmed histologically [46]. Also a recent study by Lavecchia et al. reported that a cavity anterioposterior distance of more than 15 mm was associated with the need for D&C and an unplanned return to the emergency department [47]. However, another study by Creinin et al. showed a wide range of endometrial thickness (1–31 mm) 2 weeks after expulsion of the gestational sac and a decreasing endometrial thickness over time. The authors suggest that clinical signs and symptoms should guide treatment decisions after medical treatment [48]. Expectant management in case of an endometrial thickness more than 15 mm 1 week after medical treatment is advised on the basis of recent findings by the Dutch nationwide MisoREST-study, [15, 16] The MisoREST-study investigated whether curettage is more effective than expectant management in case of an incomplete evacuation (sonographic endometrial thickness > 10 mm) 1 week after misoprostol treatment concludes that expectant management until 6 weeks after medical treatment is safe, effective in approximately 80% of patients, and that women have a clear preference for expectant management instead of curettage [15].

In our trial, ultrasonography will be performed between day 15 and 20 to evaluate the first treatment effect (Fig. 1). In case of an expulsed gestational sac, and an total endometrial thickness (TED) < 15 mm by ultrasonography, no further evaluation is necessary and treatment is considered as successful. In case of expulsed sac but possible retained products of conception (TED > 15 mm) expectant management is advised, with consent from the patient, for another 4 weeks. Patients are able to contact their hospital 24 h a day in case of any questions, complaints or emergencies. During these weeks of expectant management, clinical signs and symptoms should determine whether additional treatment (e.g. curettage) may be necessary. If successful curettage has been performed after medical treatment, no further examinations for the purposes of the study project are necessary. Six weeks after treatment, ultrasonography will be performed to evaluate endometrial thickness. In case of an endometrial thickness > 15 mm 6 weeks after treatment, further treatment will be according to local protocol and patient preferences. Additional treatment may be expectant, medical or surgical (hysteroscopy or D&C).

Anti-D prophylaxis will be given if necessary as part of the standard treatment, following the NVOG-guideline “Erytrocytenimmunisatie en zwangerschap” [49].

### Outcome measures

Primary and secondary outcome measures will be extracted from routine clinical parameters in the patient medical record and patient diary and recorded in a digital case report form. A two-step method will be used to determine treatment success. Ultrasonography will be performed 2 weeks after medical treatment to determine treatment failure defined as a complete gestational sac intrauterine. The definite primary study outcome, complete (success) or incomplete (failure) evacuation, will be determined 6 weeks after treatment [2, 8, 15, 16, 46, 48, 50, 51]. A successful medical treatment will be considered in case of an ultrasonography showing a TED < 15 mm (maximum anterior-posterior diameter, two or 6 weeks after medical treatment) and no evidence of retained products of conception using only the allocated therapy by randomisation.

Secondary outcomes include patient satisfaction, complications, side effects and costs. Other interventions such as urgent surgical curettage, the need for blood transfusion, additional hospital admissions or late interventions such as hysteroscopy will also be reported. Secondary outcome measures are subtracted from the medical record, patient diary and (validated) digital questionnaires. At baseline, day five, and two and 6 weeks after treatment started, questionnaires will be sent by email. To measure the quality of the health status of the patients, two so-called health-related quality of life (HRQoL) instruments will be used: the Short Form 36 health survey and the EuroQol-5D, both available in a Dutch translation. Patient preferences and satisfaction with treatment will be measured using The Client Satisfaction Questionnaire (CSQ-8, digital) two and 6 weeks after treatment. In order to enable a thorough cost-effectiveness analysis patients will also receive the iMTA Productivity Cost Questionnaire (iPCQ) with questions regarding their ability to perform work and with that their productivity loss.

### Economic evaluation

A cost-effectiveness analysis will be performed, from a societal perspective. To evaluate which medical treatment strategy is cost-effective, volumes of health care consumed will additionally be measured prospectively alongside the clinical trial together with cost associated with productivity losses. Costs of medical interventions (direct costs) and costs resulting from productivity loss (indirect costs) will be taken into account. Resource uses will be recorded in the case report forms. Standardised unit costs will be calculated using the Dutch manual for costing in economic evaluations.

### Statistical issues

#### Sample size calculation

Based on retrospective data in the Radboud University Medical Centre (Nijmegen) that are compatible with data from the literature, we found a complete evacuation rate in the control group in 54 and 67% in the intervention group [21]. We used these rates for the calculation of the sample size with an overall significance level of 5%, α = 0.05, in combination with a power of 80%, β = 0.20. Based on an improvement of complete evacuation rates from 54 to 67%, the trial requires 221 patients in each arm. Considering 3–4% patients lost-to-follow-up, 230 patients per arm have to be included (total 460).

Because of the intended execution of an interim analysis, the sample size will have to be adjusted to maintain a sufficient powered final analysis. This leads to a total number of 464 (1.008*460 = 463,68) required inclusions, 232 per arm.

#### Data analysis

Data handling will be done anonymized, with the patient code only available to the treating physician and local investigator. Data will initially be analysed according to intention to treat method. The main outcome variable will be assessed by calculating success rates in both groups, relative risks, and 95% confidence intervals. A per protocol analysis will be performed to evaluate the potential of both strategies, taking into account only those cases that were treated according to protocol. Differences between groups will be analysed using the Pearson’s chi-square test or the Fisher’s exact test for categorical variables and the Students t-test for continuous variables. Mann-Whitney U test will be used for non-normally distributed metric variables and univariate and multivariate logistic regression analysis to identify individual factors that are associated with treatment success. Economic analysis will be done according to intention to treat principle. Differences in total costs between the intervention and control group will be calculated.

#### Interim analysis and safety monitoring

A data safety monitoring board has been established. The DSMB is independent of the study organisers. After including 50 % of the anticipated patients in each arm, an interim analysis will be done using O’Brien-Fleming stopping rules. In light of this interim analysis, the DSMB will advice the researchers if, in it’s view the active intervention has been proven (beyond reasonable doubt) to be different from the control for all or some types of participants, and if the evidence on the economic outcomes is sufficient, to guide a decision from health care providers regarding recommendation of the sequential use of mifepristone and misoprostol. This means that if mifepristone followed by misoprostol is particularly beneficial or harmful compared to the control group, the investigators will be able to make a deliberate consideration of terminating the study earlier. Local investigators will report (serious) adverse events as soon as possible to the sponsor. The sponsor is responsible to report serious adverse events (SAE’s) within 15 days to the ethical committee Commissie Mensgebonden Onderzoek Arnhem-Nijmegen.

## Discussion

Yearly in the Netherlands, approximately 10.000 women with EPF do not abort spontaneously and, after a minimum of 1 week of expectant management, undergo medical or surgical treatment in order to remove the products of conception from the uterus. Medical treatment is a proven safe and less expensive alternative to D&C. However, since there is no national guideline describing the treatment options for EPF, there is a large practice variation between Dutch hospitals [34].. The current medical treatment with misoprostol after a minimum of 1 week of expectant management results in a complete evacuation rate of around 50%. Thus, 50% of women may still be exposed to the risks of complications and costs associated with surgery [2, 14–16].

Medical treatment for EPF may be improved by pre-treatment with mifepristone followed by the current treatment with misoprostol alone. The superiority of this sequential combination has been demonstrated for termination of vital pregnancy in the first trimester, preparation for surgical abortion in the first trimester, termination of vital pregnancy beyond first trimester, and induction of labour in case of fetal death after the first trimester [17, 52, 53]. Therefore, it is reasonable to consider that also for EPF mifepristone followed by misoprostol will be superior to misoprostol alone.

A randomised, double blinded placebo-controlled trial is required to deliver the ultimate evidence that in EPF the sequential combination of mifepristone with misoprostol is superior to the use of misoprostol alone with respect to complete evacuation of products of conception, side effects, complications, patient preferences, and costs. Shortly after the start of this study an article about the PreFaiR trial was published, showing an advantage to mifepristone pretreatment [54]. To deliver irrefutable evidence to clinicians all over the world, we believe it is relevant to vouch these outcomes, in which a placebo-controlled comparison will provide the most impeccable results.

## Supplementary information

**Additional file 1.** List of ethics approval of participating study sites of the Triple M Trial.

## Data Availability

Not applicable.

## Abbreviations

D&C: Dilatation and curettage
EPF: Early pregnancy failure
M&M: Mifepristone + misoprostol
TED: Total endometrial thickness
