# Supplementary material for: Mifepristone and misoprostol versus misoprostol alone for uterine evacuation after early pregnancy failure: study protocol for a randomized double blinded placebo-controlled comparison (Triple M Trial)
Source: BMC Pregnancy Childbirth. 2019 Nov 27;19:443. doi: 10.1186/s12884-019-2497-y (PMC6880504; doi:10.1186/s12884-019-2497-y)
Supplement: Supplementary file 1 — Additional file 1. List of ethics approval of participating study sites of the Triple M Trial. [file 12884_2019_2497_MOESM1_ESM.docx]

**List of ethics approval of participating study sites of the Triple M Trial**

The authors declare that in addition to assessment and approval of the Medical Ethical Committee and the Competent Authorithy the following councils have assessed and approved participation of their hospital in the Triple M Trial:

Board of Directors of Radboud University Medical Centre

Board of Directors of Canisius Wilhelmina Hospital

Board of Directors of Maasstad Hospital

Board of Directors of Haaglanden Medical Centre

Board of Directors of OLVG location East

Board of Directors of Rijnstate

Board of Directors of VieCuri Medical Centre

Board of Directors of Zuyderland Medical Centre

Board of Directors of St. Antonius Hospital

Board of Directors of Catharina Hospital

Board of Directors of Laurentius Hospital

Board of Directors of Amphia Hospital

Board of Directors of Gelre Hospitals

Board of Directors of Jeroen Bosch Hospital

Board of Directors of Hospital Gelderse Vallei

Board of Directors of Ikazia Hospital
